# Recanalization of superficial femoral artery chronic total occlusion through retrograde popliteal approach recanalization of superficial femoral artery chronic total occlusion

**DOI:** 10.1016/j.heliyon.2024.e30872

**Published:** 2024-05-09

**Authors:** Chuli Jiang, Yu Zhao, Wayne W. Zhang, Zheng Chen, Qiu Zeng, Fenghe Li

**Affiliations:** a Department of Vascular Surgery, The First Affiliated Hospital of Chongqing Medical University, China; b Division of Vascular and Endovascular Surgery, Department of Surgery, University of Washington, and Puget Sound VA Health Care System, USA

**Keywords:** Peripheral arterial disease, Flush occlusion, Superficial femoral artery, Retrograde popliteal approach, Vascular closure devices

## Abstract

**Purpose:**

This retrospective study aims to evaluate the safety, practicality, and efficacy of the independent retrograde popliteal approach for recanalization superficial femoral artery (SFA) occlusions when the bilateral common femoral artery approach is unavailable, such as after endovascular aneurysm repair or common iliac artery stenting.

**Methods:**

This treatment was considered for patients with contralateral iliac artery occlusion, severe iliac tortuosity, or those who had previously undergone endovascular aneurysm repair or common iliac stenting. Patients with SFA lesions extending into the P1–P2 segment of the popliteal artery or with calcification in the P3 segment were excluded. Angioplasty and stenting were conducted via the popliteal artery approach, with hemostasis at the puncture site achieved using an EXOSEAL vascular closure device. Patients were routinely followed up at 3, 6, and 12 months, and annually thereafter.

**Results:**

Forty-eight consecutive patients with SFA occlusion who underwent endovascular treatment via the retrograde popliteal artery approach were included in this study. Retrograde puncture of the popliteal artery was successful in all cases. Six-French sheaths were utilized in all procedures. The EXOSEAL vascular closure device was successfully applied in all 48 cases. No instances of pseudoaneurysms, arteriovenous fistulas, major bleeding, or embolic complications were observed. The technical success rate for SFA recanalization was 100 %. All patients experienced clinical improvement. The ankle-brachial index significantly increased from an initial 0.33 ± 0.11 at admission to 0.81 ± 0.19 at discharge (P < 0.001). The mean follow-up period was 25.1 ± 11.7 months. Kaplan-Meier analysis revealed primary patency rates of 82.5 % at 12 months and 71.8 % at 24 months. No patients required major amputation during the follow-up period.

**Conclusion:**

The endovascular treatment of SFA occlusions via the independent retrograde popliteal approach is a viable alternative, demonstrating a low incidence of puncture-related complications and a high success rate of recanalization.

## 1 Introduction

Peripheral arterial disease significantly impacts population health, with profound implications for both morbidity and mortality. Globally, 202 million people were living with peripheral arterial disease. The superficial femoral artery (SFA) is regarded as the most common diseased vessel in peripheral arterial disease [1]. A substantial proportion of peripheral atherosclerotic lesions be detected in the SFA, appearing as diffuse lesions or chronic total occlusions (CTO) [2]. Endovascular techniques have become the standard for managing complex SFA lesions, with successful treatment depending on appropriate access. The transfemoral approach is preferred, utilizing the common femoral artery (CFA) for either antegrade or retrograde catheterization based on the lesion's location. The contralateral retrograde approach is advisable for flush SFA occlusions and tandem iliofemoral lesions. In rare instances, such as after previous endovascular aneurysm repair or common iliac artery stenting, when neither bilateral CFA is viable for access, the popliteal artery may serve as an alternative approach for treating SFA lesions [3].

Previously, few studies have explored stent placement via the popliteal artery approach for SFA lesions, primarily due to concerns about the risk of bleeding at the popliteal artery puncture site. The deep location and the loose surrounding tissue of the popliteal artery puncture site have always raised concerns regarding hemostasis [4]. Previous literature indicates that hematoma incidence could reach up to 10 % with the insertion of only a 4 French sheath [5]. These concerns have limited attempts to use larger sheaths for angioplasty or stenting via the popliteal artery approach. There is limited experience with using larger sheaths (≥6 French) for the retrograde popliteal artery approach. The vascular closure device (VCD) is an effective strategy in achieving CFA puncture site hemostasis [6,7], and a few studies have demonstrated the potential of VCDs for popliteal artery access hemostasis [8,9]. This opens the possibility of using larger sheaths in the popliteal artery for angioplasty or stent placement. If necessary, SFA recanalization could be performed via an independent retrograde popliteal approach, with VCDs facilitating puncture site hemostasis. However, the literature on this specific application remains sparse. This study presents an initial experience with SFA revascularization employing the independent retrograde popliteal approach.

## 2 Methods

### 2.1 Patient population

Between December 2017 and February 2023, a total of 48 consecutive patients who underwent endovascular treatment for femoral occlusion via retrograde popliteal artery access were retrospectively included. This group comprised patients with flush occlusion of the SFA, those with contralateral iliac artery occlusion, severe iliac tortuosity, and those who had previously undergone endovascular aneurysm repair or common iliac stenting. Patients with SFA lesions extending into the P1–P2 segment of the popliteal artery or with calcification in the P3 segment were excluded. We reviewed demographics, indications for transpopliteal access, Rutherford classification, TASC II classification, and risk factors for atherosclerosis in all patients.

### 2.2 Procedures

The procedure was carried out in the angio-suite under local anesthesia. The patients were placed supine with the lower extremity in a 60° external rotation and the knee in a mild flexion. Retrograde ipsilateral popliteal artery puncture was performed under ultrasound guidance using the Micropuncture Acess Set (Cook Medical, Bloomington, Ind). Popliteal vessels with no significant calcification were chosen for a puncture. A 6 French sheath (Cordis, Bridgewater, NJ, USA) was introduced after a successful puncture confirmed by ultrasound (Supplemental Fig. 1). Then, 5000 IU of heparin was administered intravenously once placement of the vascular sheath was achieved.

**Fig. 1:**
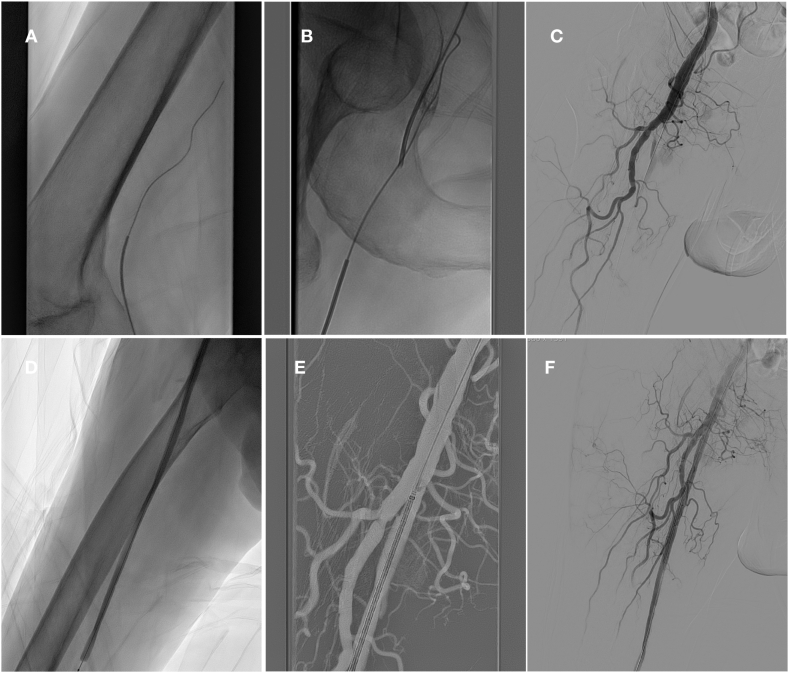
Images showing the steps of recanalization of SFA lesion through the retrograde popliteal approach. (A) The target lesion was traversed with guidewire via the popliteal arterial approach. (B) A through-and-through technique was used to pass the targeted lesion. (C) An angiography was performed via the brachial arterial approach to show the extent of the lesion. (D) Balloon dilatation. (E) Stenting via transpopliteal approach. (F) Angiogram showed good ﬂow within the stent.

The target lesion was traversed with a 0.018-inch wire (V18™ Control Wire, Boston Scientific™, MA, USA) and vertebral catheter (Cordis, Bridgewater, NJ, USA) via the popliteal arterial approach. If the passage of the lesion was difficult, the brachial artery was punctured using the Micropuncture Access Set (Cook Medical, Bloomington, Ind) with a 4F sheath inserted. The 4 French vertebral catheter (Cordis, Bridgewater, NJ, USA) and 0,035-inch hydrophilic guidewire (Terumo, Tokyo, Japan) were descended via the brachial artery approach, and a through-and-through technique was used to pass the targeted lesion. Once crossing the lesion successfully, an angiogram was performed to ensure the catheter was in the true lumen. And then, balloon angioplasty was performed. Self-expanding stents were deployed through the popliteal artery approach to cover the target lesion (Fig. 1A–F). The technical success was defined as a successful popliteal artery puncture and a patent SFA with <30 % residual stenosis. After the interventional procedure, the puncture site was sealed with an EXOSEAL VCD (Cordis, Miami Lakes, Florida), constituting an off-label use of the device (Supplemental Fig. 2). After the VCD application, manual compression was applied to the puncture site for 5 min. A compression bandage was placed over the puncture site and kept in situ for 6 h. Postoperatively, Aspirin 100 mg once daily and rivaroxaban 2.5 mg twice daily were administered.

### 2.3 Follow-up

Ultrasound evaluation of the puncture site was c conducted 24 h post-intervention. Follow-ups were scheduled at 3, 6, and 12 months, and annually thereafter, including physical examinations, duplex ultrasound of target vessels, and ankle-brachial index measurements. Primary patency was defined as a >50 % reduction in vessel diameter on DUS, with a peak systolic velocity ratio <2.4. Computer tomography angiography was performed for significant restenosis detected by duplex ultrasound.Statistical Analysis.

Continuous data were analyzed for normal distribution with the Kolmogorov–Smirnov test. Continuous data were presented as mean ± standard deviation and compared using Student's t-test. A P value of <0.05 was considered statistically significant. Categorical data were presented as the count (percentage). The Kaplan-Meier method was applied to estimate patency, survival, and target lesion revascularization (TLR) rates. The SPSS software package, version 25.0 (SPSS Software Inc, Chicago, IL) was used for statistical analysis.

## 3 Results

From December 2017 to February 2023, 3215 patients underwent endovascular interventions for lower extremity arteriosclerosis obliterans at our institution. Among these, 2147 had flush occlusion of the SFA. A subset of these patients, including 20 with contralateral iliac artery occlusion, 19 with severe iliac tortuosity, and 9 who had previously undergone EVAR or common iliac stenting, were selected for percutaneous recanalization via the retrograde popliteal approach. In total, 48 patients underwent this procedure. The average age of these patients was 73.7 ± 6.8 years, with an average BMI of 21.1 ± 1.7 kg/m^2^. The majority were men (41/48, 85.4 %) and smokers (39/48, 81.2 %). The average lesion length was 261.31 ± 57.46 mm, with most lesions classified as TASC D (38/48, 79.2 %). Baseline characteristics of the patients are detailed in Table 1.

**Table 1:** Baseline Clinical Characteristics of the patients.

Variables	N = 48
Age, years	73.7 ± 6.8
BMI, kg/m^2^	21.1 ± 1.7
Men	41(85.4)
ABI	0.32 ± 0.11
Mean lesion length, mm	261.31 ± 57.46
Indications for transpopliteal access	
Contralateral iliac artery occlusion	20(41.6 %)
Severe iliac tortuosity	19(39.5 %)
Previously EVAR or iliac stent	9(18.9 %)
TASC II classification	
C	10(20.8 %)
D	38(79.2 %)
Rutherford classification	
III	25(52.1 %)
IV	16(33.3 %)
V	5(10.4 %)
VI	2(4.2 %)
Comorbidities	
Smoking	39(81.2 %)
Diabetes mellitus	17(35.4 %)
Hypertension	22(45.8 %)
Hyperlipidemia	9(18.7 %)
Coronary artery disease	11(22.9 %)

ABI: ankle-brachial index. TASC:TransAtlantic Inter-Society Consensus. Continuous data are presented as the means ± standard deviation; categorical data are given as the counts (percentage).

Under ultrasound guidance, retrograde popliteal artery puncture was successfully performed in all cases, with a 6F sheath inserted at the puncture site for all 48 patients. SFA recanalization was technically successful in every case, with two patients requiring an additional brachial artery puncture for a through-and-through technique. Balloon angioplasty was standard, and stenting stenting was requisite in the majority (43/48, 89.5 %). Bare metal stents were employed exclusively, with specific product usage distributed as follows: EverFlex (Medtronic, Plymouth, MN, USA) in 26 patients, Innova (Boston Scientific, Marlborough, MA, USA) in 14 patients, and Eluvia (Boston Scientific, Marlborough, MA, USA) in 3 patients. The average stent length was 279.34 ± 42.22 mm, with diameters ranging from 4 to 7 mm, the most common being 6 mm (Table 2). Drug-coated balloons were utilized in 8 (15.7 %) patients.

**Table 2:** Procedure-related information.

Variables	N = 48
Technical success	48(100 %)
Recanalization methods	
DCB	8(16.7 %)
Stenting	43(89.5 %)
Types of stents	
EverFlex	26(60.6 %)
Innova	14(32.6)
Eluvia	3 (6.8 %)
Mean stent length, mm	279.34 ± 42.22
Labeled stent diameter,mm	
4.0	3
5.0	16
6.0	19
7.0	5
Access-related complication	
Hematoma	5(10.4 %)
Major bleeding	0(0)
Pseudoaneurysm	0(0)
Arteriovenous fistula	0(0)
Distal embolization	0(0)

Continuous data are presented as the means ± standard deviation; categorical data are given as the counts (percentage).

Closure of the popliteal artery puncture site with the EXOSEAL VCD (Cordis, Bridgewater, NJ, USA) was successful in all patients, with no instances of distal embolization during or subsequent to the sealing process. Duplex ultrasound detected small hematomas (<1 cm) at the popliteal puncture site in five patients 24 h post-procedure, all of which were asymptomatic, and which resolved spontaneously without intervention. There were no reports of major bleeding, pseudoaneurysm, arteriovenous fistula, or popliteal vein thrombosis.

**Fig. 2:**
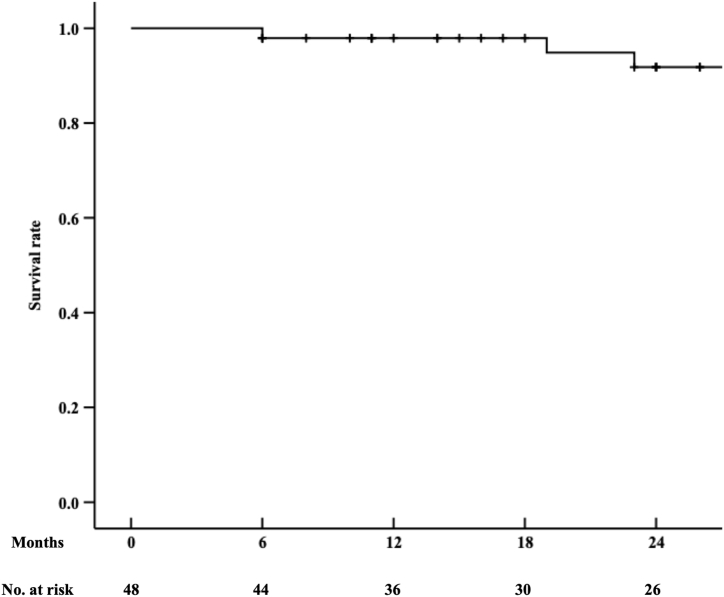
Kaplan–Meier survival curves were used to estimate survival rate of patients. The survival rates at 24 months were 91.8 %.

**Fig. 3:**
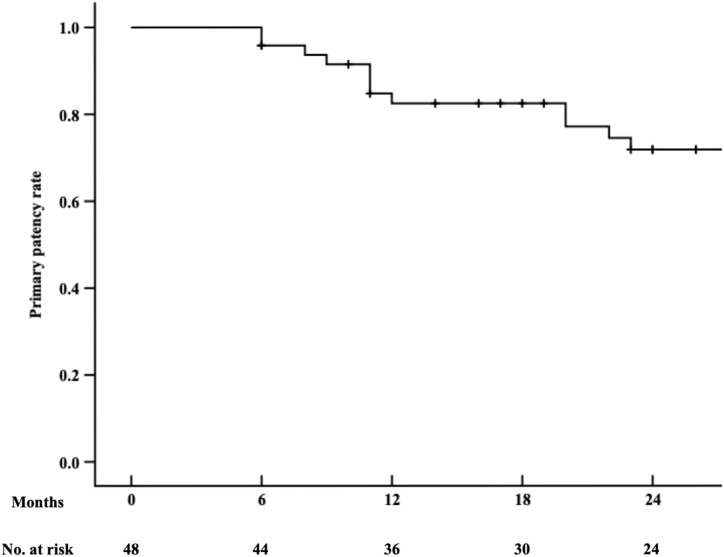
Kaplan–Meier analysis of primary patency rate. The primary patency rate was 82.5 % and 71.8 % at 12 and 24 months, respectively.

**Fig. 4:**
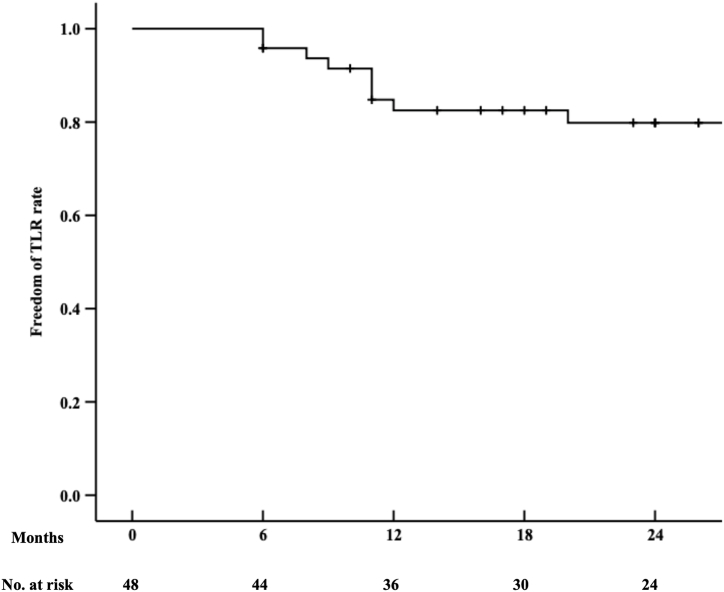
Kaplan–Meier analysis of freedom from target lesion revascularization (TLR) rate. The freedom from TLR rate at 12 months and 24 months was 82.5 % and 79.8 % respectively.

The mean follow-up period was 25.1 ± 11.7 months. Three patients died of unrelated causes but remained asymptomatic in peripheral arterial disease. The Kaplan-Meier curve indicated survival rates of 91.8 % at 24 months (Fig. 2). The ankle-brachial index improved significantly from an initial 0.33 ± 0.11 at admission to 0.81 ± 0.19 at discharge, 0.86 ± 0.13 at 6 months, 0.79 ± 0.22 at 12 months, and 0.72 ± 0.17 at 24 months (P < 0.001). Clinical improvement was observed in all patients. The primary patency rate was 82.5 % at 12 months and 71.8 % at 24 months, as shown by Kaplan-Meier curves (Fig. 3). During the follow-up, 13 patients developed in-stent restenosis, and 10 underwent reinterventions for persistent symptoms. The freedom from target lesion revascularization (TLR) rate was 82.5 % at 12 months and 79.8 % at 24 months (Fig. 4). Thrombolysis was performed in four patients using a catheter placed through the brachial artery, and drug-coated balloon angioplasty was utilized in another six patients via the *trans*-popliteal approach. The secondary patency rate was 100 % during the follow-up, and no patient required major amputation.

## 4 Discussion

The retrograde popliteal access was first introduced in the 1980s [10]. Initially, it did not gain widespread acceptance due to the awkward positioning required and the risk of complications at the access site. Trigaux et al. described the popliteal artery puncture technique, emphasizing the anatomical relationship between the popliteal artery and vein [11]. Subsequently, a series of studies have demonstrated the feasibility of the popliteal artery puncture approach under ultrasound or fluoroscopy guidance, reporting favorable technical success rates [[12], [13], [14], [15], [16]]. After failed antegrade attempts, the retrograde approach was sometimes required to pass the guidewire through the lesion. Several studies have highlighted the effectiveness of the retrograde approach, in conjunction with CFA access, in recanalizing SFA occlusions [13,17,18]. This method has increasingly become an essential adjunct to CFA access. In rare cases, when the CFA is inaccessible, retrograde popliteal artery access may be the only approachable vessel in the lower extremities. The results of 48 patients indicated that the retrograde popliteal access resulted in a high technical success rate with rare complications under ultrasound guidance and with the assistance of the EXOSEAL VCD. Only four small, asymptomatic hematomas were noted in the popliteal region, and all patients experienced successful SFA recanalization with satisfactory 24-month patency rates.

Our study confirms the promising technical success rate of ultrasound-guided retrograde popliteal artery puncture, aligning with prior research [19]. Ultrasound guidance enhances the precision of the popliteal artery puncture, reduces the likelihood of vasospasm from multiple puncture attempts, and minimizes the risk of accidental venous injury and arteriovenous fistula formation. Moreover, it allows practitioners to circumvent calcified plaques, which could otherwise result in hematomas post-procedure.

The hemostasis of the popliteal artery puncture site has been a concern, especially after a more prominent sheath placement [13,16]. While earlier studies predominantly used 4F sheaths for popliteal access [3], our findings indicate that the EXOSEAL VCD is both safe and effective for achieving hemostasis after inserting a 6 French sheath. In the present study, a 6 French sheath was necessary for balloon angioplasty and stent placement, with EXOSEAL VCD application recommended for all patients to ensure proper hemostasis. No major bleeding, pseudoaneurysm, arteriovenous fistula or thrombo-embolization were observed in our cohort, with only four small hematomas detected by follow-up ultrasound and no instances of popliteal vein thrombosis. Currently, several VCDs are available besides EXOSEAL. Angio-Seal employs a collagen plug with intravascular components to aid in hemostasis. StarClose VCD, a clip-type device, does not have intravascular components but necessitates the use of a specific sheath during application. Some previous literature has reported major complications related to these VCDs. In Noory's study, 28 cases underwent popliteal artery closure with StarClose VCD [20]. During sheath removal, one popliteal occlusion (3.5 %) was found in the form of an atherosclerotic plaque shift caused by StarClose VCD. In another study by Noory et al., focusing on retrograde transpopliteal recanalization in 56 patients, popliteal artery stenosis caused by an intravascular component of Angio-Seal was noted in one patient and subsequently managed with stent placement [17]. Distinct from the above VCD, EXOSEAL can be used to avoid the potential damage to the popliteal artery by changing sheaths. Additionally, its application of an extravascular plug, devoid of intravascular components, minimizes the risk of thrombosis linked to intravascular anchors. The design benefits of EXOSEAL contribute to its safety in achieving hemostasis at popliteal artery puncture sites.

SFA lesions are often diffuse and severe, typically classified as TASC II C or D, making endovascular treatment challenging. In our group, SFA recanalization was achieved in all patients, demonstrating a promising success rate for transpopliteal recanalization of SFA CTO lesions. Kuserli reported on 93 patients who underwent balloon dilation via popliteal retrograde access, achieving a technical success rate of 92.47 % [19]. Dumantepe conducted recanalization of SFA CTO in 28 subjects through the retrograde popliteal approach, with endoluminal recanalization successful in 26 (92.8 %) patients, indicating the popliteal approach's effectiveness in facilitating SFA CTO lesion recanalization [21]. Several factors contribute to why these lesions may be more amenable to retrograde crossing. Firstly, the distal side of the plaque is usually softer than the proximal cap, potentially allowing easier wire passage [15,22,23]. Additionally, since the initial cap has a smooth surface and a convex shape, the guidewire from the top may slide past the cap into the subintimal space. The guidewire can be kept in the center of the vessel lumen from the popliteal artery to make it easier for crossing retrogradely [24]. Moreover, the short distance between the access site and the occluded segment provides a greater push ability for the wire and catheter [19]. In our group, primary patency rates at 12, 24 months were 82.5 % and 71.8 %, respectively. In the previous studies, reported primary patency at one year ranged from 45.1 % to 86 % [15,17,25,26]. Considering most patients in our study had TASC D SFA occlusions, the procedure's durability is deemed acceptable. All patients in the current study experienced clinical improvement, and none required major amputation during the follow-up period.

Our study has demonstrated that the use of VCD at the popliteal artery puncture site is safe, and interventional treatment via the popliteal artery approach can effectively manage SFA lesions. This research opens up additional avenues for addressing complex SFA lesions. Nevertheless, the study is limited in several aspects. First, this is a retrospective study with relatively small sample size. Second, selection bias is inevitable since all patients undergoing SFA recanalization through the popliteal approach had to meet specific, predetermined criteria. The complexity of the lesions in these patients exceeds that typically seen in the broader patient population, which may influence the long-term outcomes of the procedure. Furthermore, the follow-up period was relatively short, and long-term follow-up is required to understand the durability of SFA recanalization via the retrograde popliteal artery approach. Future large-scale, multicenter, and prospective studies are needed to further validate our results.

## 5 Conclusion

The results of this study suggest that when the CFA approach was inaccessible, endovascular treatment of SFA lesions via the retrograde popliteal approach is a feasible alternative technique, which can be safely performed by an experienced operator. Accessing the retrograde popliteal artery under ultrasound guidance is a secure and viable method. The use of the EXOSEAL VCD can further minimize the risks of bleeding and hematoma formation at the puncture site. The percutaneous recanalization conducted through the popliteal approach is linked with a high rate of technical success.

## Ethics statement

The study was approved by the ethical commission of Ethics Committee of the First Affiliated Hospital of Chongqing Medical University (approval number: 2023-064-01). All participants provided written informed consent for the publication of all images, clinical data and other data included in the manuscript.

## Data availability

Data associated with the study has not been deposited into a publicly available repository. Data are available from the corresponding author on reasonable request.

## CRediT authorship contribution statement

**Chuli Jiang:** Writing – review & editing, Writing – original draft, Resources, Project administration, Methodology, Investigation, Formal analysis, Data curation, Conceptualization. **Yu Zhao:** Writing – review & editing, Resources, Project administration, Methodology, Investigation, Formal analysis, Data curation, Conceptualization. **Wayne W. Zhang:** Resources, Project administration, Methodology, Investigation, Formal analysis, Data curation, Conceptualization. **Zheng Chen:** Visualization, Validation, Project administration, Methodology, Investigation, Formal analysis, Data curation, Conceptualization. **Qiu Zeng:** Visualization, Methodology, Investigation, Formal analysis, Data curation, Conceptualization. **Fenghe Li:** Writing – review & editing, Visualization, Validation, Resources, Project administration, Methodology, Investigation, Formal analysis, Data curation, Conceptualization.

## Declaration of competing interest

The authors declare that they have no known competing financial interests or personal relationships that could have appeared to influence the work reported in this paper.
