# Supplementary material for: Recanalization of superficial femoral artery chronic total occlusion through retrograde popliteal approach recanalization of superficial femoral artery chronic total occlusion
Source: Heliyon. 2024 May 9;10(11):e30872. doi: 10.1016/j.heliyon.2024.e30872 (PMC11140595; doi:10.1016/j.heliyon.2024.e30872)
Supplement: Multimedia component 1 [file mmc1.docx]

**
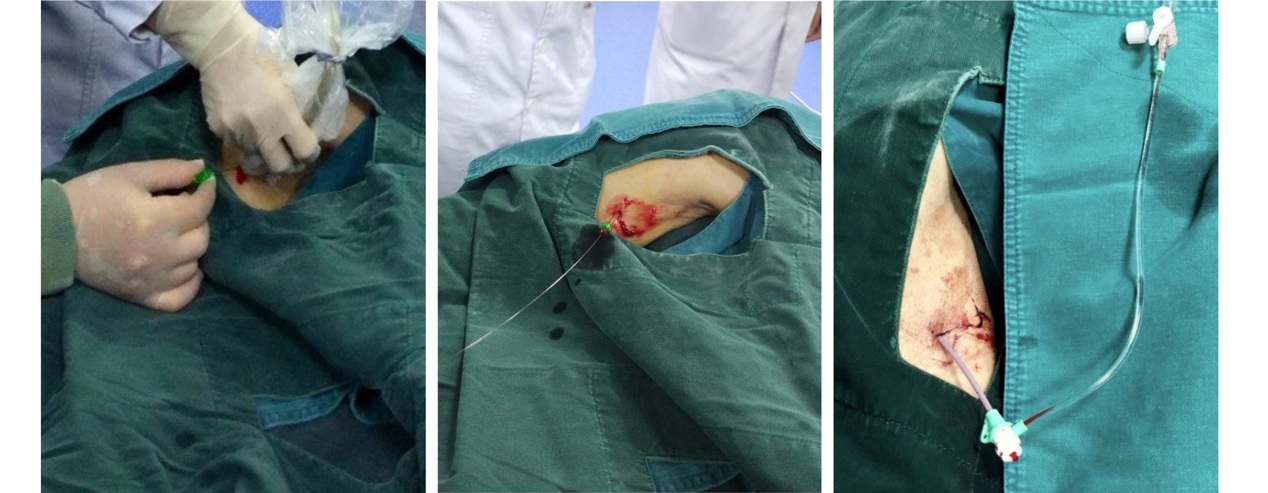
**

**Supplemental Figure.1** Retrograde popliteal artery puncture was performed under ultrasound guidance and a 6F sheath was introduced after a successful puncture.


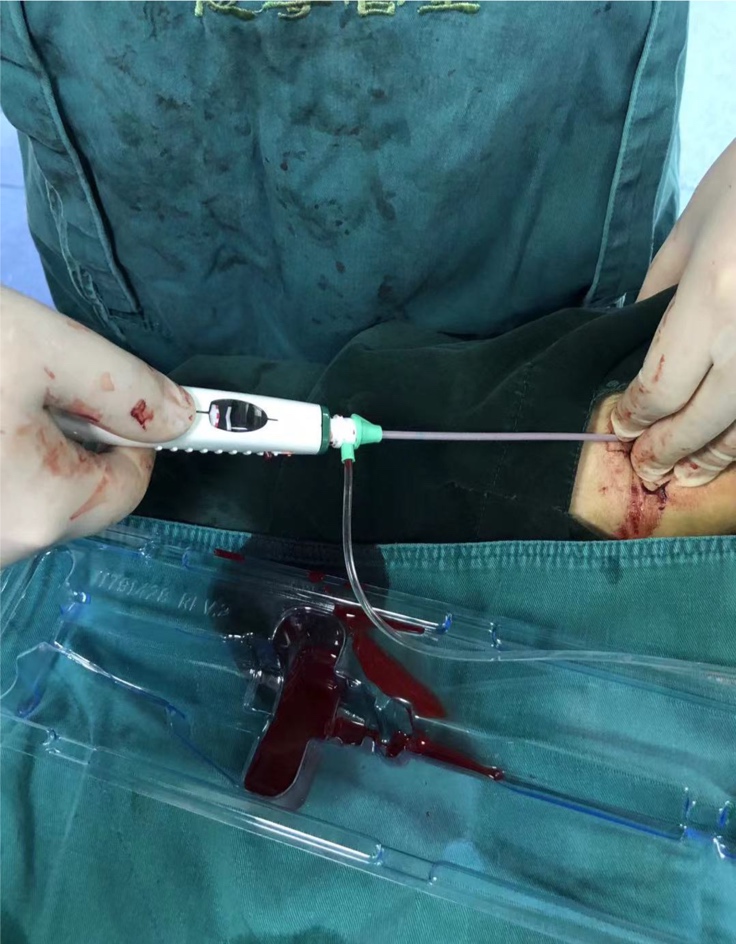


**Supplemental Figure.2** Sealing the puncture site with an EXOSEAL.
